# Impacts of the COVID-19 Pandemic on the Global Agricultural Markets

**DOI:** 10.1007/s10640-020-00473-6

**Published:** 2020-08-04

**Authors:** Christian Elleby, Ignacio Pérez Domínguez, Marcel Adenauer, Giampiero Genovese

**Affiliations:** 1 European Commission, Joint Research Centre, Seville, Spain; 2 Agro-Food Trade and Markets Division, OECD, Paris, France

**Keywords:** COVID-19, GDP shock, Agricultural markets, Biofuel markets, Aglink-Cosimo

## Abstract

This paper analyses the impacts on global agricultural markets of the demand shock caused by the COVID-19 pandemic and the first wave of lockdown measures imposed by the governments in the first semester of 2020 to contain it. Specifically, we perform a scenario-based analysis on the IMF economic growth forecasts for 2020 and 2021 using a global multi-commodity agricultural market model. According to our results, the sharp decline in economic growth causes a decrease in international meat prices by 7–18% in 2020 and dairy products by 4–7% compared to a business as usual situation. Following the slowdown of the economy, biofuel prices fall strongly in 2020, followed by their main feedstocks, maize and oilseeds. Although the income losses and local supply chain disruptions associated with the pandemic undoubtedly has led to an increase in food insecurity in many developing countries, global food consumption is largely unaffected due to the inelastic demand of most agricultural commodities and the short duration of the shock. From an environmental viewpoint, the COVID-19 impacts point to a modest reduction of direct greenhouse gases from agriculture of about 1% or 50 million tonnes of carbon dioxide equivalents in 2020 and 2021.

## Introduction

The COVID-19 pandemic, which has led to the loss of more than 500 thousand lives out of 10.3 million confirmed cases (as of June 30, 2020), has also caused a global downturn comparable, by some measures, to that of the great depression in the 1930s. The causes of the two economic crises are, however, very different and it is also believed that the recovery from the current crisis will be faster than the recovery from the great depression.

There is still a lot of uncertainty though, as to how long the COVID-19 recession will last and what the global economic consequences will be in the medium term. It depends on a number of factors affecting supply and demand of all good including agricultural commodities. These include, how quickly businesses around the world will be able to re-open from the lockdowns; whether there will be secondary waves forcing governments to impose new lockdown measures; how soon a vaccine and/or an effective treatment against the SARS-CoV-2 virus is available and how all this affect consumer spending patterns. Nonetheless, there are already several global economic outlooks that account for the COVID-19 impact in their GDP projections. The IMF, World Bank and OECD forecasts for the global GDP contraction in 2020 are in the range 3.0–7.5% and the forecasts for the ensuing global GDP increase in 2021 range from 2.8 to 5.8% (World Bank 2020; IMF 2020; OECD 2020a). Building on the IMF forecast, the International Food Policy Research Institute (IFPRI) estimates that the economic contraction in 2020 could increase the number of people living in extreme poverty by a staggering 20% or 140 million people, which will result in a heightened level of food insecurity in many countries (Laborde et al. 2020).

In this paper, we add to the growing literature on the economic effects of the COVID-19 pandemic with an analysis of the impacts on the global agricultural commodity markets. The pandemic has reminded us just how dependent we are on a well-functioning global food value chain and how vulnerable we are to disruptions in this key sector. A sudden lack of mobility across borders and within countries has caused labour shortages in countries that are reliant on seasonal migrant workers in the agri-food sector, which, in turn, has affected food availability and prices globally (FAO 2020). In India and in several African countries, for example, the price of several key staples have reportedly increased by more than 15% as from pre-COVID-19 levels (Hernandez et al. 2020).^1^

The pandemic has also affected trade of goods through e.g. additional border controls, lack of cargo shipments and reinforced sanitary controls. Moreover, similar to the 2007–2008 food crisis, the pandemic led some countries to impose export restrictions in order to protect their domestic consumers (WTO 2020). Such trade frictions could also affect global food prices.

Due to the lack of data, we do not consider these supply-side disruptions to the agri-food sector in this paper. Instead, we focus on the demand shock caused by a general loss of income affecting consumers’ spending patterns. The resulting lower demand obviously leads to a downward pressure on producer prices and production, but it is not clear, a priori, how large the effect will be in the different interdependent agricultural sectors. The meat sector, for example, is directly affected by lower demand for meat products resulting from lower incomes and by substitution towards cheaper (plant based) sources of calories. However, lower demand for grain and oilseeds also reduces feed costs, so the size of the net effect on production and prices is unclear.

In our discussion of the COVID-19 impacts, we have a particular focus on the biofuel markets. These markets have been especially affected by the pandemic because, of the lockdowns in many countries, which have driven down the demand for transport fuel. Moreover, the resulting fall in international oil prices has made biofuels less competitive with fossil fuels. Lower demand for biofuels affects the demand for its feedstocks, maize and oilseeds, which, in turn, affect the markets for other crops and animal products.

One positive side-effect of the drop in consumption of fuel and the general disruption of economic activity in connection with the pandemic, is a significant decrease in global greenhouse gas (GHG) emissions (Le Quéré et al. 2020; Rugani and Caro 2020). For many years, the ‘degrowth’, movement has been arguing for the need to reduce consumption in order to reduce the ecological footprint of human activities (Georgescu-Roegen 1977; Kerschner 2010). More recently, in connection with the adoption of the Paris agreement in 2015, governments around the world have committed to reducing their GHG emissions through national policies in order to limit the global temperature increase to well below 2 °C (Schleussner et al. 2016). In the EU, specifically, the European Commission (EC) launched its ‘Green Deal’ in 2019, which aims to have zero net emissions of greenhouse gases in 2050 and where economic growth is decoupled from resource use.^2^

The agricultural sector is an important contributor to global GHG emissions and the sector, therefore, faces a societal pressure to reduce its climate impact (IPCC 2019; Schiermeier 2019; Wollenberg et al. 2016). In the EU, the EC put forward its legal proposal in 2018 for the implementation of the Common Agricultural Policy (CAP) for the period 2021–2027.^3^ The proposal introduces a new programming tool call national ‘Strategic Plans’, giving the Member States more freedom to choose and implement policies that can meet the objectives of the future CAP, one of which is ‘climate change action’. These Strategic Plans must reflect the ambition of the Green Deal and are assessed against climate and environmental criteria.^4^ In the proposed budget for the Multi-Annual Financial Framework, 25% of the Direct Payments Budget is allocated to Eco–Schemes, 30% of the Rural Development funds are allocated to Agro-environmental and Climate Measures^5^ and Voluntary Coupled Support is maximized including the additional 2% of Pillar I for protein crops.

In late May, the EC presented its COVID-19 recovery package containing a reinforced EU budget for 2021–2027 as well as a Recovery Instrument called ‘Next Generation EU’, which will allow the EC to borrow up to EUR 750 billion on the financial markets.^6^ Included in this is a proposal to increase funding for the European Agricultural Fund for Rural Development by EUR 15 billion and to strengthen the Just Transition Fund up to EUR 40 billion in order to accelerate the transition towards climate neutrality and to support the changes required to achieve the targets in the Green Deal.

In light of these requirements for the agricultural and other sectors to reduce their climate footprint, and the considerable effort it takes to make this happen, it is interesting to see how the pandemic affects agricultural GHG emissions. We therefore quantify the COVID-19 impact on GHG emissions associated with the agricultural production changes.

The paper now proceeds as follows. Section 2 describes the model, database and assumptions going into the calculations. Section 3 discusses the scenario results and Sect. 4 offers concluding remarks.

## Methodology

### The Model

The scenario analysis in this paper is based on a set of simulations carried out with the Aglink-Cosimo model. Aglink-Cosimo is a recursive-dynamic partial equilibrium model developed and maintained by the Organisation for Economic Co-operation and Development (OECD) Secretariat and the Food and Agriculture Organization of the United Nations (FAO) as a collaborative effort (OECD 2015; Araujo-Enciso et al. 2015). The model is used to produce the OECD-FAO and EU Medium Term Agricultural Outlooks, yearly publications aiming to provide baseline projections for the main agricultural commodities over the medium term (OECD-FAO 2019; EC 2019). These deterministic baseline projections are accompanied by a partial stochastic analysis that considers yields, international oil prices and several macroeconomic variables as stochastic variables (Araujo-Enciso et al. 2017).

### Scenario Design and Main Assumptions

**Fig. 1:**
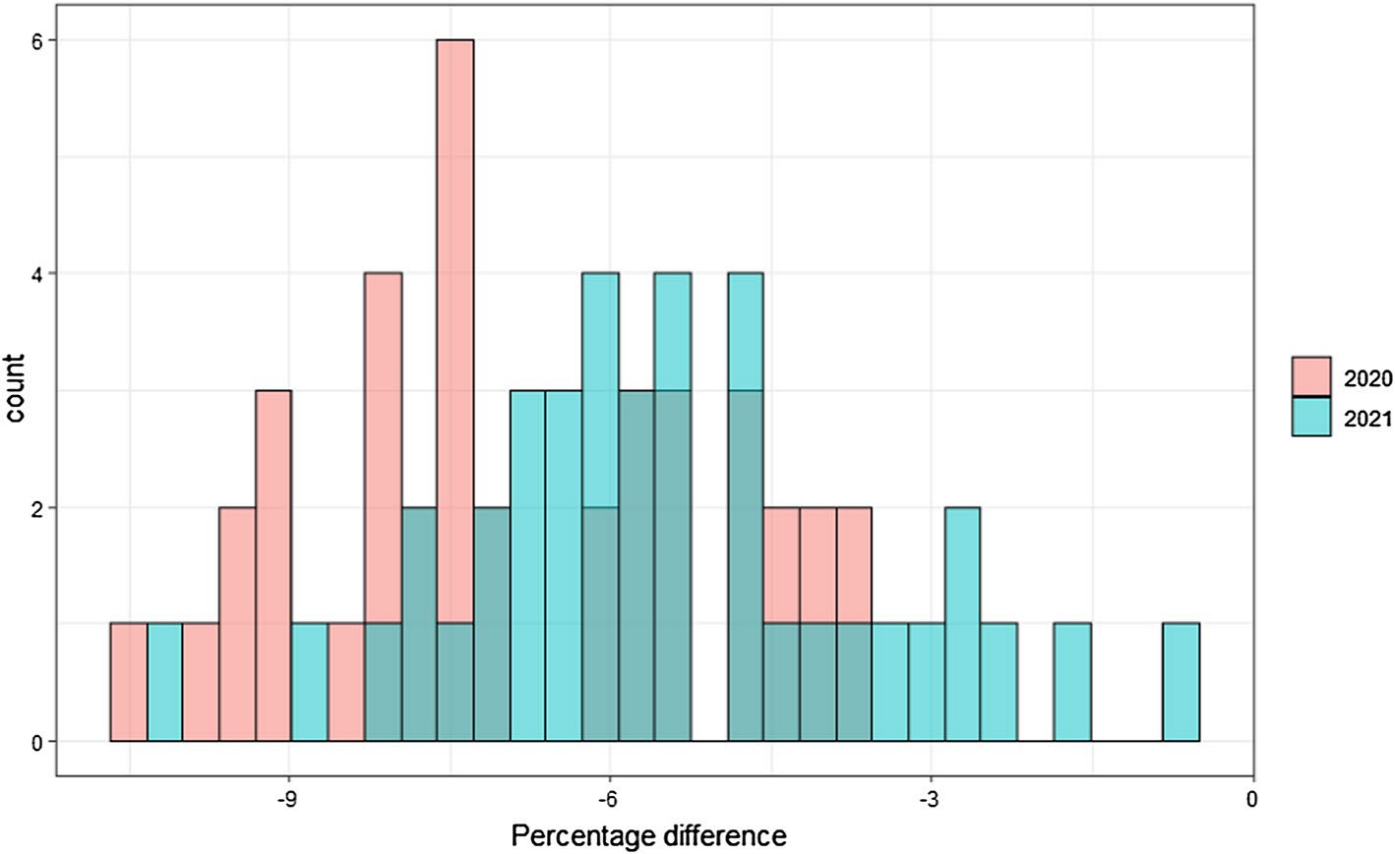
Real GDP. Percentage difference between Baseline and Scenario

We analyse a single scenario based on country specific GDP growth forecasts from the IMF World Economic Outlook database (April 2020), supplemented with EU figures from the Spring 2020 Economic Forecast by the European Commission. The scenario shocks are the forecasted GDP growth rates for 2020 and 2021, applied to 2020 GDP baseline and implied scenario value for 2021, respectively. From 2022 and onwards, we assume that the GDPs return to their baseline values.^7^ Fig. 1 illustrates the difference in baseline and scenario GDP values. The baseline is the OECD-FAO Agricultural Outlook 2019–2028 (OECD-FAO 2019; OECD 2020b) extended to 2030.

In addition to the scenario impacts, expressed as a set of point estimates, we also carry out a stochastic analysis focused on the relationship between international oil prices and agricultural commodity markets. Agricultural and energy markets are interrelated mainly through the production of biofuels (mandated largely) and input costs (e.g. fertiliser costs). Currently we are facing a period of low oil prices, due to a combination of supply and demand factors. However, as discussed below, international oil prices are exogenous in the model and we have not made any assumptions about their deviation from the baseline in order to keep a strict focus on the demand effects of COVID-19. Instead, we quantify the joint distribution of scenario impacts, where the variation comes from alternative oil price projections, based on the historical variation in oil prices.

## Results

### Global Impacts

**Fig. 2:**
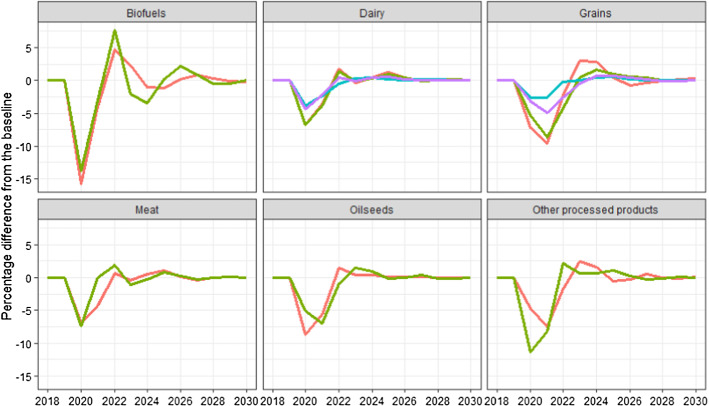
Impacts on world prices. Percentage difference from the baseline. Same commodities as in Table 1

Table 1 summarises the estimated impacts on global food prices until 2025, from the GDP shocks in Fig. 1. In 2020, prices of agricultural commodities and derived products decrease sharply, especially biofuels, meats and vegetable oils. In 2021, the prices are still below their baseline value except for some of the meats (pork and sheep). In 2022 the picture is more mixed, with grains and biofuels being above the baseline and others still below. When we reach 2025, all of the commodity prices are close to their baseline values and, as illustrated in Fig. 2, this continues to be the case until the end of the projection period in 2030.

**Table 1:** Impacts on world prices. Percentage difference from the baseline

	2020	2021	2022	2023	2024	2025	Band (%)
*Biofuels*							
Biodiesel	− 15.9	− 4.0	4.7	2.3	− 1.0	− 1.2	22.4
Ethanol	− 13.8	− 3.1	7.7	− 2.1	− 3.5	0.2	13.6
*Dairy*							
Butter	− 6.8	− 3.6	1.7	− 0.3	0.4	1.3	4.6
Cheese	− 6.8	− 3.9	1.4	− 0.1	0.4	1.0	3.0
Skim milk powder	− 3.8	− 2.4	− 0.4	0.3	0.5	0.2	0.9
Whole milk powder	− 4.4	− 2.3	0.4	0.0	0.4	0.6	2.8
*Grains*							
Maize	− 7.2	− 9.7	− 2.3	3.0	2.8	0.4	6.6
Other coarse grains	− 5.3	− 8.7	− 4.3	0.4	1.6	1.0	3.6
Rice	− 2.7	− 2.7	− 0.3	0.0	0.4	0.5	2.7
Wheat	− 3.2	− 4.9	− 2.6	− 0.4	0.7	0.7	3.9
Meat							
Beef and Veal	− 10.4	− 0.5	6.9	− 1.5	− 3.0	0.9	1.8
Pork	− 17.6	4.7	7.2	− 4.3	− 0.8	2.9	3.7
Poultry	− 7.0	− 4.2	0.7	− 0.3	0.5	1.1	3.2
Sheep	− 7.3	0.0	1.9	− 1.1	− 0.3	0.8	1.8
*Oilseeds*							
Other Oilseeds	− 8.7	− 5.7	1.5	0.4	0.5	0.1	7.8
Soybean	− 5.1	− 6.9	− 1.0	1.5	0.9	− 0.1	6.6
*Other processed products*							
Total Protein Meal	− 4.7	− 7.5	− 1.7	2.5	1.6	− 0.5	4.5
Vegetable oils	− 11.4	− 8.1	2.2	0.7	0.7	1.1	6.6

The column (Band  %) is a measure of the variation in world production resulting from stochastic oil prices. It represents the 95% range (the large bands in Fig. 4) as a percent of the scenario value in 2030

Table 2 summarises the global production impacts corresponding to the price changes in Table 1. As expected, the demand shock leads to lower production, but the reduction is obviously much lower than for prices, due to the inelastic demand for agricultural commodities. Also, many production decisions for 2020 where already taken when the pandemic started so that production effects in 2021 are stronger than in 2020 for most commodities.

**Table 2:** Impacts on world production. Percentage difference from the baseline

	2020	2021	2022	2023	2024	2025	Band %
*Biofuels*							
Biodiesel	− 2.1	− 2.1	− 1.0	− 0.3	0.1	0.1	3.0
Ethanol	− 3.0	− 3.1	− 0.5	0.2	0.2	0.0	3.1
*Dairy*							
Butter	− 1.0	− 1.1	− 0.2	0.2	0.1	− 0.1	0.1
Cheese	− 2.5	− 1.4	0.3	0.0	− 0.1	0.0	0.1
Skim milk powder	− 1.3	− 1.1	0.1	− 0.2	− 0.1	0.0	0.2
Whole milk powder	− 1.7	− 0.6	0.0	− 0.1	− 0.1	0.0	0.2
*Grains*							
Maize	0.0	− 1.0	− 1.5	− 0.7	0.2	0.6	1.6
Other coarse grains	0.0	− 0.1	− 0.5	− 0.4	− 0.1	0.1	0.4
Rice	0.0	− 0.1	− 0.1	0.0	0.0	0.0	0.3
Wheat	0.0	0.0	− 0.2	− 0.2	− 0.2	− 0.1	0.8
*Meat*							
Beef and Veal	0.1	− 1.5	− 2.0	0.2	1.1	− 0.2	0.3
Pork	− 0.3	− 2.0	− 0.5	0.5	− 0.1	− 0.3	0.3
Poultry	− 2.1	− 0.5	1.0	− 0.1	− 0.5	0.1	0.3
Sheep	− 0.9	− 0.8	0.1	0.2	0.0	0.0	0.1
*Oilseeds*							
Other Oilseeds	0.0	− 1.2	− 1.0	0.0	0.2	0.2	1.0
Soybean	0.0	− 0.5	− 1.0	− 0.4	0.2	0.3	1.6
*Other processed products*							
Total Protein Meal	− 0.5	− 0.8	− 0.4	− 0.1	0.0	0.0	0.6
Vegetable oils	− 0.3	− 0.8	− 0.5	− 0.1	0.0	0.1	0.5

The column (Band  %) is a measure of the variation in world production resulting from stochastic oil prices. It represents the 95% range (the large bands in Fig. 4) as a percent of the scenario value in 2030

### Regional Impacts

Domestic markets are related to the world market through trade. Therefore, a price increase on the world market will typically cause an increase in the domestic price as well. Tables 3 and 4 summarise the domestic price impacts in four large countries/regions, Brazil, China, EU and USA, in order to get a sense of the variation across countries.

**Table 3:** Impacts on EU and US producer prices. Percentage difference from the baseline

	EU				USA			
	2020	2021	2022	Band (%)	2020	2021	2022	Band (%)
*Biofuels*								
Biodiesel	–14.7	− 2.6	3.9	60.7	–20.4	− 7.9	6.1	107.2
Ethanol	–11.7	− 1.2	6.8	36.9	–	− 3.9	9.0	48.8
*Dairy*								
Butter	− 4.1	− 2.4	1.3	55.2	− 7.5	− 4.6	2.0	36.6
Cheese	− 4.3	− 2.3	0.7	11.5	− 7.4	− 4.6	1.6	10.6
Skim milk powder	− 3.4	− 1.9	− 0.7	20.1	− 3.9	− 2.5	− 0.4	16.8
Whole milk powder	− 3.9	− 2.0	0.3	5.7	− 4.6	− 2.6	0.3	5.5
*Grains*								
Maize	− 1.0	− 1.9	− 0.8	11.5	− 7.3	− 9.7	− 2.2	12.2
Other coarse grains	− 3.6	− 6.4	− 2.9	5.7	− 5.2	− 8.5	− 4.4	5.8
Rice	− 2.7	− 2.7	− 0.3	56.6	− 2.7	− 2.8	− 0.3	125.0
Wheat	− 3.2	− 5.0	− 2.5	6.1	− 3.1	− 4.9	− 2.8	6.0
*Meat*								
Beef and Veal	–10.4	− 0.8	5.7	17.9	–21.3	− 3.7	22.0	15.5
Pork	–18.5	4.6	9.4	16.4	–21.0	1.7	12.8	18.8
Poultry	− 7.6	− 3.6	0.9	38.6	− 7.2	− 4.3	0.7	60.4
Sheep	− 7.6	0.1	2.1	38.2	− 8.5	− 0.6	2.8	28.1
*Oilseeds*								
Other Oilseeds	− 8.7	− 4.6	2.6	13.3	− 8.4	− 6.0	0.9	12.4
Soybean	− 5.1	− 6.8	− 0.9	9.9	− 5.0	− 7.0	− 1.1	10.4
*Other processed products*								
Total Protein Meal	− 4.2	− 6.7	− 1.6	37.2	− 4.7	− 7.4	− 1.8	28.0
Vegetable oils	–13.1	− 8.7	2.4	16.4	–11.7	− 8.4	2.3	15.0

The columns (Band  %) is a measure of the variation in world production resulting from stochastic oil prices. It represents the 95% range (the large bands in Fig. 4) as a percent of the scenario value in 2030

**Table 4:** Impacts on China and Brazil producer prices. Percentage difference from the baseline

	China				Brazil			
	2020	2021	2022	Band (%)	2020	2021	2022	Band (%)
*Biofuels*								
Biodiesel	− 5.1	− 0.7	0.1	91.3	− 8.7	− 6.1	3.6	162.7
Ethanol	− 7.9	− 0.7	6.8	52.4	–12.0	− 3.1	6.6	89.6
*Dairy*								
Butter	− 9.9	− 4.3	1.7	60.9	–19.7	− 4.8	5.8	141.4
Cheese	− 6.7	− 2.9	0.9	72.6	–11.7	− 6.6	3.1	250.1
Skim milk powder	− 3.9	− 2.0	− 0.4	44.8	4.1	− 8.2	− 2.8	79.1
Whole milk powder	− 4.3	− 1.6	0.3	32.8	− 3.9	− 5.9	0.3	48.0
*Grains*								
Maize	− 3.6	− 4.9	− 1.0	59.4	− 7.2	− 9.4	− 1.9	137.3
Other coarse grains	− 5.3	− 8.4	− 4.3	53.7	− 6.0	–10.2	− 6.4	109.7
Rice	− 0.4	− 0.4	− 0.2	40.2	− 3.0	− 2.4	− 0.2	64.7
Wheat	− 1.0	− 1.7	− 1.2	62.7	− 3.7	− 5.0	− 2.3	160.9
*Meat*								
Beef and Veal	–	3.3	15.1	31.5	− 9.2	− 0.6	5.9	45.0
Pork	–14.7	4.2	4.2	63.8	–18.6	4.3	7.5	168.3
Poultry	− 4.0	− 2.3	− 1.0	37.0	− 6.7	− 4.6	0.2	57.3
Sheep	− 7.8	0.6	0.6	16.3	− 5.8	− 4.3	0.8	19.2
*Oilseeds*								
Other Oilseeds	− 8.5	− 5.2	1.7	77.0	− 8.6	− 6.1	0.5	284.9
Soybean	− 5.1	− 6.9	− 1.1	67.8	− 5.1	− 6.8	− 0.7	186.9
*Other processed products*								
Total protein meal	− 4.5	− 7.0	− 1.6	54.0	− 4.7	− 7.4	− 1.6	110.6
Vegetable oils	–	− 8.0	2.4	61.9	–12.1	− 8.5	2.1	143.7

The columns (Band  %) is a measure of the variation in world production resulting from stochastic oil prices. It represents the 95% range (the large bands in Fig. 4) as a percent of the scenario value in 2030

As can be seen, the domestic price impacts follow the same patterns as the world prices, with the largest impacts to be found amongst the meats and dairy products, biofuels and biofuel feedstocks. Note, however, that the variation in the impacts caused by oil price volatility is much higher than in the world price case, especially for the biofuels case. More on this below.

### Energy Price Uncertainty

**Fig. 3:**
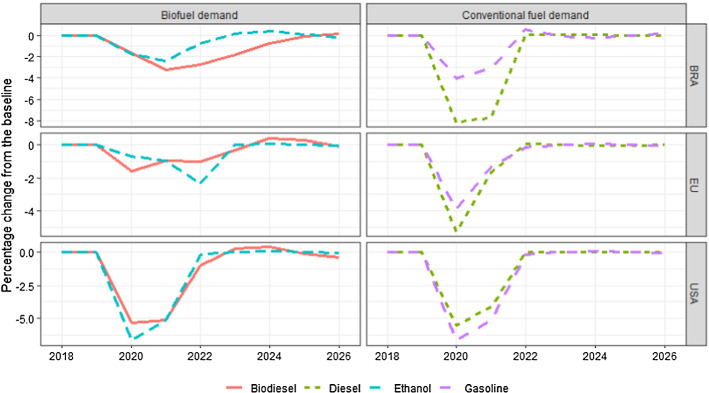
Impacts on fuel demand in Brazil, EU and USA. Percentage difference from the baseline

As Fig. 3 illustrates, transport fuel demand (incl. biofuels) reacts to changes in GDP, and fuel prices. In the three major biofuel producers US, EU and Brazil, for example, the GDP shocks lead to 4–8% lower gasoline and diesel consumption in 2020, as compared to the baseline, which, in turn, affects demand for biofuels through the blending requirements. Domestic prices of conventional fuels (gasoline and diesel), however, depends only on taxes and the exogenous world market price of oil. Therefore, in light of the recent oil price volatility, it is interesting to see how oil price volatility affects the results.

**Fig. 4:**
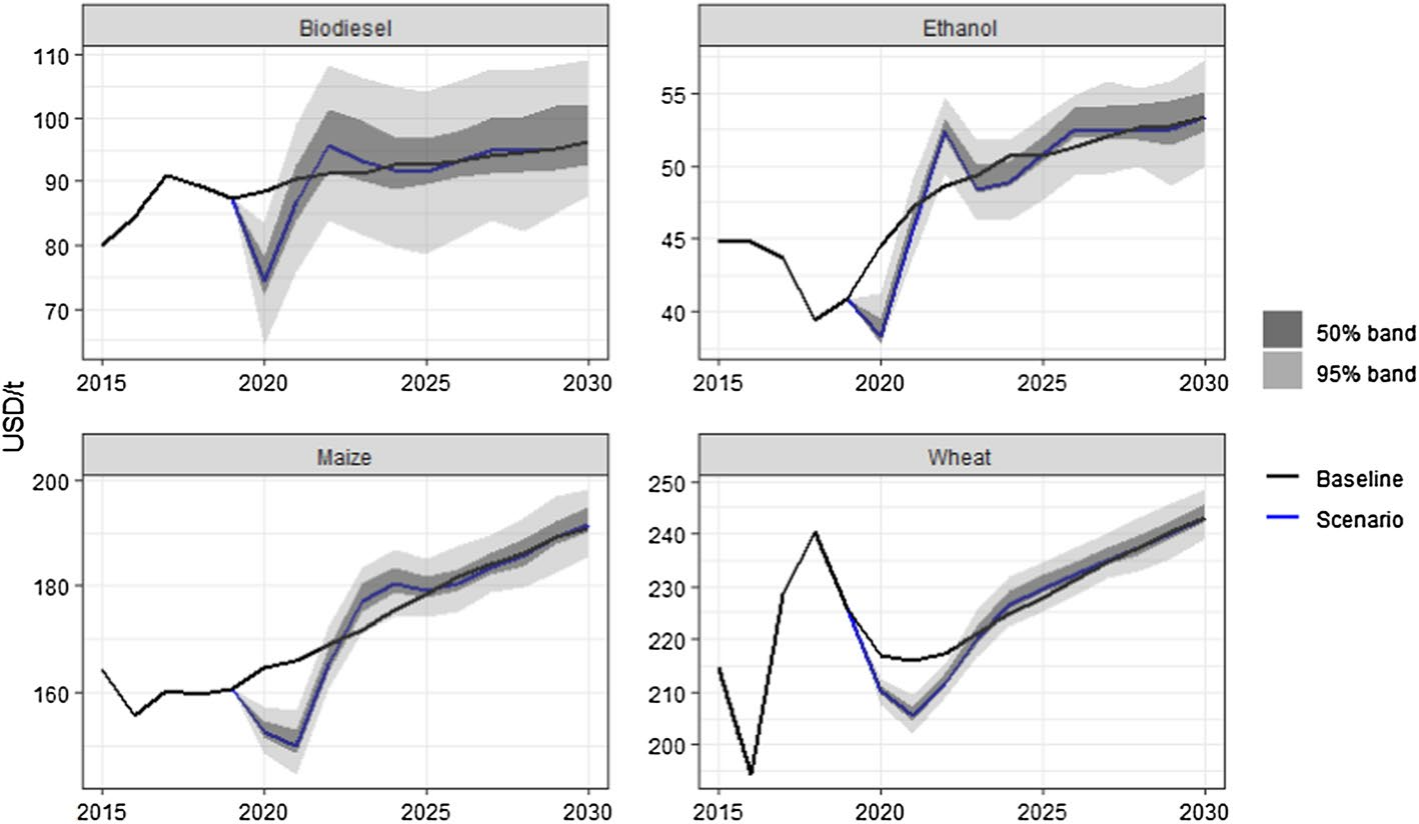
World prices of grain and biofuels in the baseline and scenario. The grey bands represent uncertainty arising from variation in international oil prices

Figure 4 illustrate how the world prices of grains and biofuels in the scenario are affected by variability in the international price of oil. Maize is a feedstock for ethanol production, so it is especially affected by oil price variability. Similarly, vegetable oil (not shown) is the major feedstock for biodiesel, so its price is also sensitive to oil price volatility.

The column labelled ‘Band  %’ in Tables 1, 2, 3 and 4 summarises how energy price volatility affect world market prices of agricultural commodities. Not surprisingly, the largest differences are for biofuels and their feedstocks (maize, oilseeds and vegetable oils). Biodiesel is the commodity that is most affected by oil prices and its 95% band in Table 1 in is equivalent to 22% of the scenario value in 2030. That is, high and low oil prices can lead to biodiesel prices that are 11% higher or lower than the one we observe in the scenario, based on the oil prices in the baseline, discarding the most extreme simulations from the upper and lower tails of the distributions. Similarly, the last column of Table 2 summarises the effects of oil price volatility on world production. Not surprisingly, the commodities with the highest dependence on oil price volatility are the same ones as in Table 1, namely the biofuels and their feedstocks (maize and oilseeds). Comparing the price bands in Tables 3 and 4 with those in Table 1, shows that the domestic price of biofuels are much more sensitive to international oil prices than their world price counterparts. This is due to biofuel policies that exists in several countries.

### Impacts on GHG Emissions

**Fig. 5:**
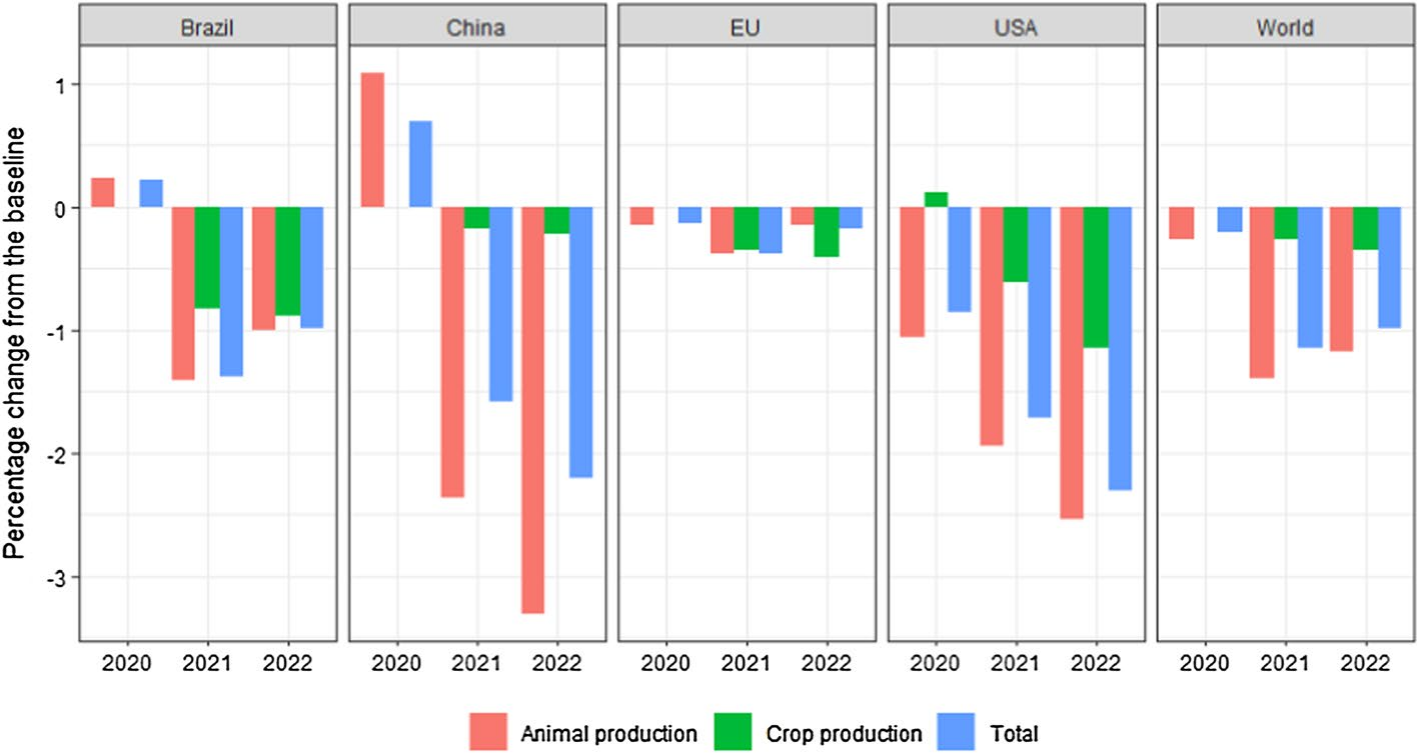
GHG emission from agricultural production. Percentage difference from the baseline

Figure 5 illustrates the estimated impact on the annual direct GHG emission from agriculture (in CO_2_ equivalents) resulting from the production changes.^8^ As one would expect, in light of the modest production changes (Table 1), the emission impacts are also modest. According to the model, the reduction in global GHG emissions in 2020–2022 are 0.2, 1.1 and 1.0%, respectively, relative to the baseline. For some of the large agricultural producers such as USA, Brazil and China, however, the reduction is larger. For China and USA, in particular, the total reduction amounts to around 2.3% in 2022 and the reduction in emissions from animal production is above 3% in 2022 for China. The lagged effects illustrate that it takes several years for the livestock sector to adjust to a demand shock.

**Fig. 6:**
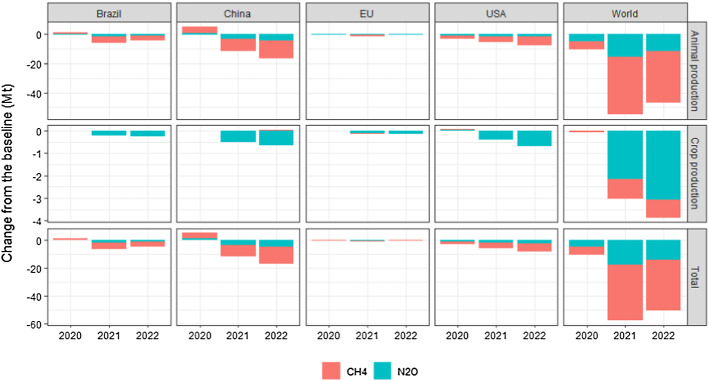
GHG emission from agricultural production. Difference from the baseline

In Fig. 6 the reduction in agricultural GHG emissions, corresponding to the percentage changes in Fig. 5, is broken down into their constituent parts, namely methane (CH_4_) emissions from enteric fermentation and nitrous oxide (N_2_O) and CH_4_ from manure. Most of the GHG reductions come from CH_4_ emission associated with ruminant production. In absolute terms, these changes correspond to more than 50 Mt of CO_2_ equivalents in 2020 and 2021.

## Conclusions

Agricultural markets are, like all other sectors, affected by the large drop in consumer spending brought on by the COVID-19 pandemic. There are already several global outlooks projecting the effects on GDP growth and other macro variables. The contribution of this paper is a quantification of the resulting impacts on the global agricultural markets and on the GHG emissions resulting from agricultural production.

Our analysis shows that the economic recession exerts downward pressure on prices, especially for high-value added commodities such as meat products and dairy. The most affected commodities are, however, biofuels and to some extent their feedstocks (e.g. maize in the US and rapeseed in Europe). Demand for these commodities is strongly linked to the demand for transport fuel, but it is also sensitive to changes in the oil price, which affects their competitiveness.

Food consumption is generally quite inelastic and it takes several years for production to adjust fully to a price change, so the GDP shocks only have a modest impact on global production and consumption. The commodities whose production change the most are the high value added products such as meat and dairy, as well as biofuels.

The modest global production changes resulting from the COVID-19 demand shock implies that the effect on global GHG emissions is also modest, around 1% in 2020–2021. However, for some of the large producers the emission reductions, especially from animal production, are in the order of 2–3%. In absolute terms, these changes correspond to around 50 Mt of CO_2_ equivalents in 2020 and 2021. From a climate policy perspective, the modest impacts on agricultural GHG emissions might seem disappointing. However, it is important to bear in mind that the scenario we consider does not include the effect of the European Green Deal or any other policies that were not implemented in 2019. Such policies that affect production and consumption incentives in the long-term, have the potential for an impact that is much greater than a (hopefully) short-term disruption like the COVID-19 pandemic.

There are several caveats to this analysis that ought to be mentioned. First, the analysis would benefit from a careful consideration of the disruptions to the supply chain brought on by the pandemic, which, reportedly, has resulted in food price increases in many countries. Although we do not model this, it is clear that a scenario with negative production/supply shocks and a reduction in exports by the main grain exporters, in addition to the negative GDP shocks, would lead to less negative price changes and it is possible that there would be price increases as well. An increase in the price of food in a situation where incomes are falling is obviously a major problem for low-income net consumers of food. For this reason, it is crucial to have reliable, quantifiable information about the magnitude of the supply disruptions caused by the pandemic, but the creation of such a database is beyond the scope of this paper.

Another improvement to the paper would be to consider several different GDP projections depending on whether additional infection waves occur in different countries. Finally, we could broaden the stochastic analysis to include macro variables and yields in order to account more fully for the uncertainty inherent in the results. We leave these refinements to future work.

## Footnotes

1 The exact causes of these price changes are unclear at this point.

2 COM(2019) 640 final.

3 COM(2018) 392; COM(2018) 393; and COM(2018) 394.

4 SWD(2020) 93 final.

5 This 30% does not include payments for Areas with Natural Constraints.

6 COM/2020/442 final; COM/2020/456 final. See also Verwey, Langedijk, & Kuenzel (2020)

7 Alternatively, we could have assumed that GDPs will follow a path parallel to their baseline values from 2022 and onwards, such that they remain below the baseline throughout the entire outlook period. Another option would be to let the GDPs converge gradually towards the baseline value at the end of the projection period. A priori, it is not obvious, which of these approaches that is the most appropriate, so we decided to go with the simplest of these for this paper.

8 Note that the emission impacts from changes to transport fuel consumption are not included in these figures.
